# Eosinophilic Pleural Effusion: A Rare Manifestation of Hypereosinophilic Syndrome

**DOI:** 10.1155/2009/635309

**Published:** 2010-01-12

**Authors:** Ndubuisi C. Okafor, Ayodeji A. Oso, Amanke C. Oranu, Steven M. Wolff, John J. Murray

**Affiliations:** Department of Internal Medicine, Meharry Medical College, Nashville, TN 37208, USA

## Abstract

Several causes of eosinophilic pleural effusions have been described with malignancy being the commonest cause. Hypereosinophilic syndrome (HES) is a rare disease and very few cases have been reported of HES presenting as eosinophilic pleural effusion (EPE). We report a case of a 26-year-old male who presented with shortness of breath. He had bilateral pleural effusions, generalized lymphadenopathy, splenomegaly, and leukocytosis with marked peripheral blood eosinophilia. The pleural fluid was exudative, with 25%–30% eosinophilis, and absence of neoplastic cells. Hypereosinophilic syndrome was diagnosed after other causes of eosinophilia were excluded. He continued to be dyspneic with persistent accumulation of eosinophilic pleural fluid, even after his peripheral eosinophil count had normalized in response to treatment. This patient represents a very unusual presentation of HES with dyspnea and pleural effusions and demonstrates that treatment based on response of peripheral eosinophil counts, as is currently recommended, may not always be clinically adequate.

## 1. Introduction

Eosinophilic pleural effusion (EPE) is defined as pleural fluid with 10% or more eosinophils [1]. Eosinophilic pleural effusions are uncommon with an incidence of 7.2% of all pleural effusions [1]. The pathogenesis of EPE involves increased production of eosinophils in the bone marrow, migration to the lungs, and extended survival of the eosinophils due to impaired apoptosis by IL-5, IL-3, and GM-CSF [2]. The causes of EPE in order of frequency include malignancy (34.8%), infections (19.2%), unknown (14.1%), posttraumatic (8.9%), and miscellaneous (23%) [1].

Hypereosinophilic syndrome (HES) is defined as peripheral eosinophilia of 1.5 × 10^9^/L, evidence of end-organ involvement, and lack of evidence for other causes of eosinophilia [3]. Hypereosinophilic syndrome can be classified as Myeloproliferative HES, Lymphocytic HES, Undefined HES, Familial HES, Associated HES, and Overlap HES [3]. Treatment of EPE is based on control of the underlying disease [1] and treatment of HES is based on control of the peripheral eosinophilia [4], which did not appear appropriate for our patient.

## 2. Case Presentation

**Figure 1:**
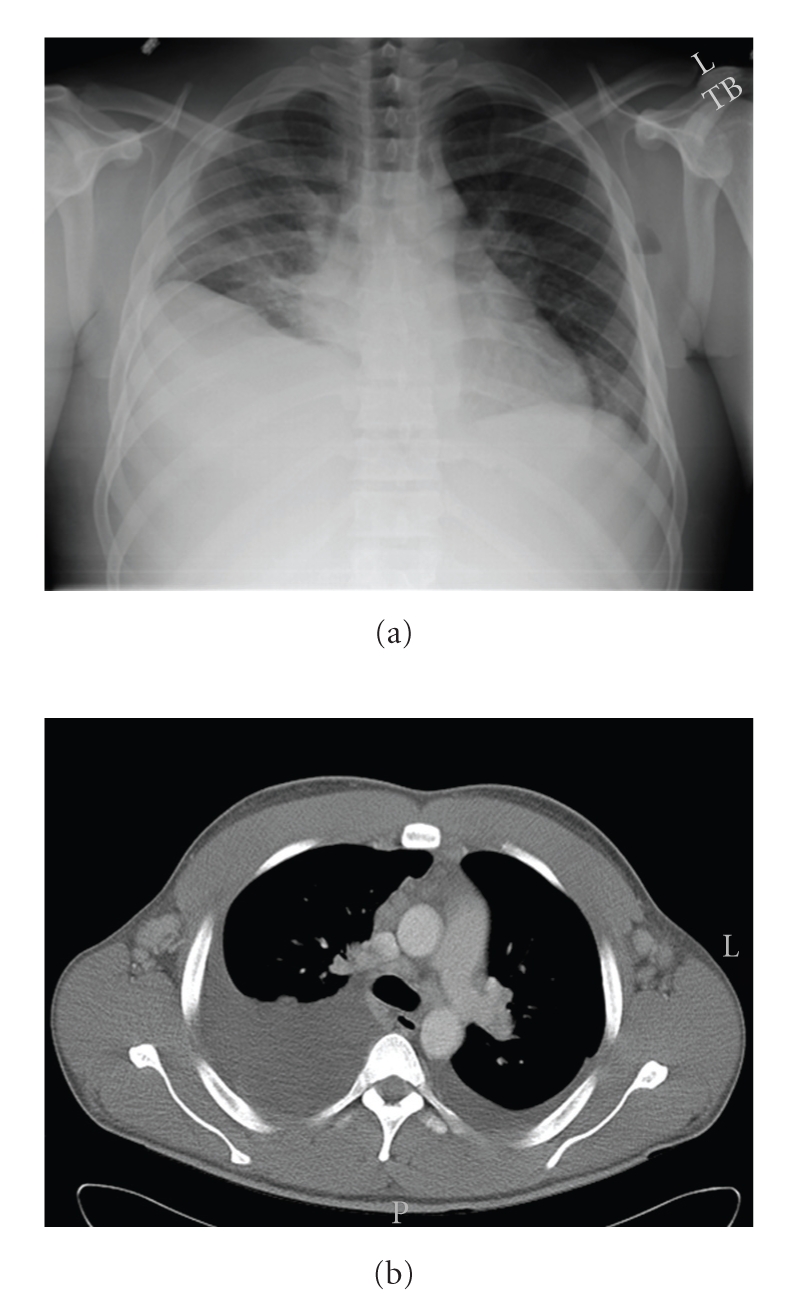
Chest radiography and CT scan of the chest showing bilateral pleural effusions.

26-year-old African American male presented with shortness of breath of over two months. He was previously healthy and had a normal CBC and differential eighteen months prior to admission. CT scan showed bilateral pleural effusions, generalized lymphadenopathy, ascites, and splenomegaly (Figure 1). Pleural fluid analysis and cytology showed an inflammatory infiltrate, reactive mesothelial cells, and 25%–30% eosinophils. No neoplastic cells were identified. Glucose, LDH, lipase, and amylase were normal. The pH was alkaline (7.53) and protein was consistent with an exudative effusion. CBC revealed a leukocytosis of 44.6 × 10^9^/L; absolute eosinophil count (AEC) was 35.6 × 10^9^/L. Peripheral blood smear, lymph node biopsy, and bone marrow aspiration showed eosinophilia with normal morphology, lymphoid hyperplasia, and hypercellular marrow with eosinophilia, respectively.

**Figure 2:**
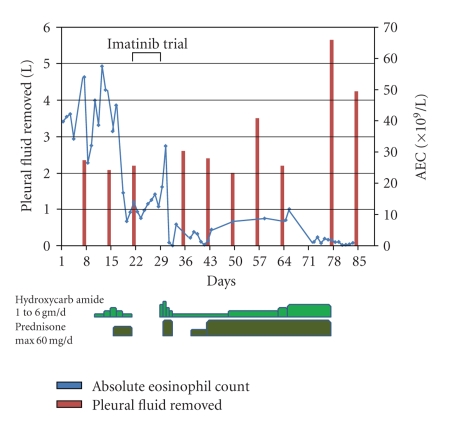
The effect of treatment on the absolute eosinophil count (AEC) in peripheral blood. Eosinophilia normalizes with hydroxycarbamide plus prednisone. Total pleural fluid removed each week was unaltered by treatment adequate to normalize peripheral eosinophil counts.

Other studies for parasitic infection, HIV, and TB were negative. Bone marrow flow cytometry showed polytypic B cells. FISH analysis and PCR assay for FIP1L1-PDGFRA fusion transcript did not detect an abnormality. A trans-thoracic echocardiography showed left ventricular ejection fraction 55%–60% with no diastolic dysfunction. A diagnosis of hypereosinophilic syndrome was made by exclusion. As initial treatment of hydroxycarbamide provided no clinical benefit or reduction in the eosinophilia, prednisone was added with prompt reduction of the eosinophilia. A trial of imatinib was given despite negative FIP1L1-PDGFRA without benefit. He had recurrent pleural effusions requiring repeated thoracentesis which continued to demonstrate persistent elevated eosinophil counts (Table 1). A permanent catheter was placed in the right pleural space and accessed for thoracentesis of about two liters every three to five days along with repeated large volume paracentesis for persistent ascites.Figure 2 shows that despite normalization of the patient's peripheral eosinophilia, his pleural effusions remained refractory to therapy. Four months after his initial presentation, he developed bilateral empyemas with methicillin resistant staphyloccus aureus as a complication of multiple thoracentesis, rapidly deteriorated in spite of aggressive management and died from multiple organ dysfunction.

## 3. Discussion

Hypereosinophilic syndrome rarely presents with eosinophilic pleural effusion. If pleural effusions are present in HES, they typically result from heart failure due to cardiac eosinophilic disease [3]. However, our patient had a normal trans-thoracic echocardiography and beta natriuretic peptide. In addition, the pleural effusion of the three previously reported patients resolved rapidly with clinical improvement, using treatment recommendations for HES of normalizing the peripheral eosinophilia [5–7]. Despite rapid reduction of the peripheral eosinophilia in our patient, the eosinophilic pleural effusion continued to accumulate for months questioning this therapeutic recommendation. A recent review of patients with EPE reported 2 out of 135 patients with percentage of eosinophils in peripheral blood higher than in pleural fluid. Those two patients had hematological malignancies [1]. Our patient had features of both the myeloproliferative and the lymphocytic variants of HES. He likely had Unclassified Complex HES. There are several chemotherapy choices depending on the variant of HES and new drugs are in clinical trials [3]. Our patient expired prior to receiving alternate treatment; however, it should be noted that the recommendation of titrating these therapies as well is normalization of the peripheral eosinophilia which had already been obtained in our patient but without clinical benefit.

## 4. Conclusion

Treatment of eosinophilic pleural effusions is directed toward the underlying cause, in this case hypereosinophilic syndrome. Disease remission cannot be determined by resolution of peripheral eosinophilia alone but also by the absence of recurrence or progression of end-organ damage [8].

## Figures and Tables

**Table 1:** Pleural fluid (PF) analysis at monthly intervals. This shows that the eosinophilia in pleural fluid persisted for months despite therapy.

	PF analysis 1	PF analysis 2	PF analysis 3
	(Day 3)	(Day 30)	(Day 64)
pH	7.53	—	—
Lipase U/L	—	<10	10
LDH U/L	94	79	458
Protein g/dL	4.88	4.61	<1.0
Glucose mg/dL	96	—	37
Amylase U/L	<10	—	<10
Nucleated cell count	1530	580	1300
Red blood cells	450	625	12640
Neutrophils	21	26	76
Mononuclear	54	41	17
Mesothelial	25	33	7
Abnormal cells	0	0	0
Culture	Negative	Negative	Staph. aureus
Cytology	25%–30% eosinophils	25% eosinophils	Primarily eosinophils
	No neoplastic cells	No neoplastic cells	No neoplastic cells

LDH: Lactate dehydrogenase.
